# Why should we care about astrocytes in a motor neuron disease?

**DOI:** 10.3389/fmmed.2023.1047540

**Published:** 2023-01-23

**Authors:** Katarina Stoklund Dittlau, Ludo Van Den Bosch

**Affiliations:** ^1^ KU Leuven—University of Leuven, Department of Neurosciences, Experimental Neurology, and Leuven Brain Institute, Leuven, Belgium; ^2^ VIB Center for Brain and Disease Research, Laboratory of Neurobiology, Leuven, Belgium

**Keywords:** amyotrophic lateral sclerosis, gain-of-toxicity, loss-of-support, disease mechanism, disease model, induced pluripotent stem cell

## Abstract

Amyotrophic lateral sclerosis (ALS) is the most common motor neuron disease in adults, causing progressive degeneration of motor neurons, which results in muscle atrophy, respiratory failure and ultimately death of the patients. The pathogenesis of ALS is complex, and extensive efforts have focused on unravelling the underlying molecular mechanisms with a large emphasis on the dying motor neurons. However, a recent shift in focus towards the supporting glial population has revealed a large contribution and influence in ALS, which stresses the need to explore this area in more detail. Especially studies into astrocytes, the residential homeostatic supporter cells of neurons, have revealed a remarkable astrocytic dysfunction in ALS, and therefore could present a target for new and promising therapeutic entry points. In this review, we provide an overview of general astrocyte function and summarize the current literature on the role of astrocytes in ALS by categorizing the potentially underlying molecular mechanisms. We discuss the current efforts in astrocyte-targeted therapy, and highlight the potential and shortcomings of available models.

## 1 Introduction

**FIGURE 1:**
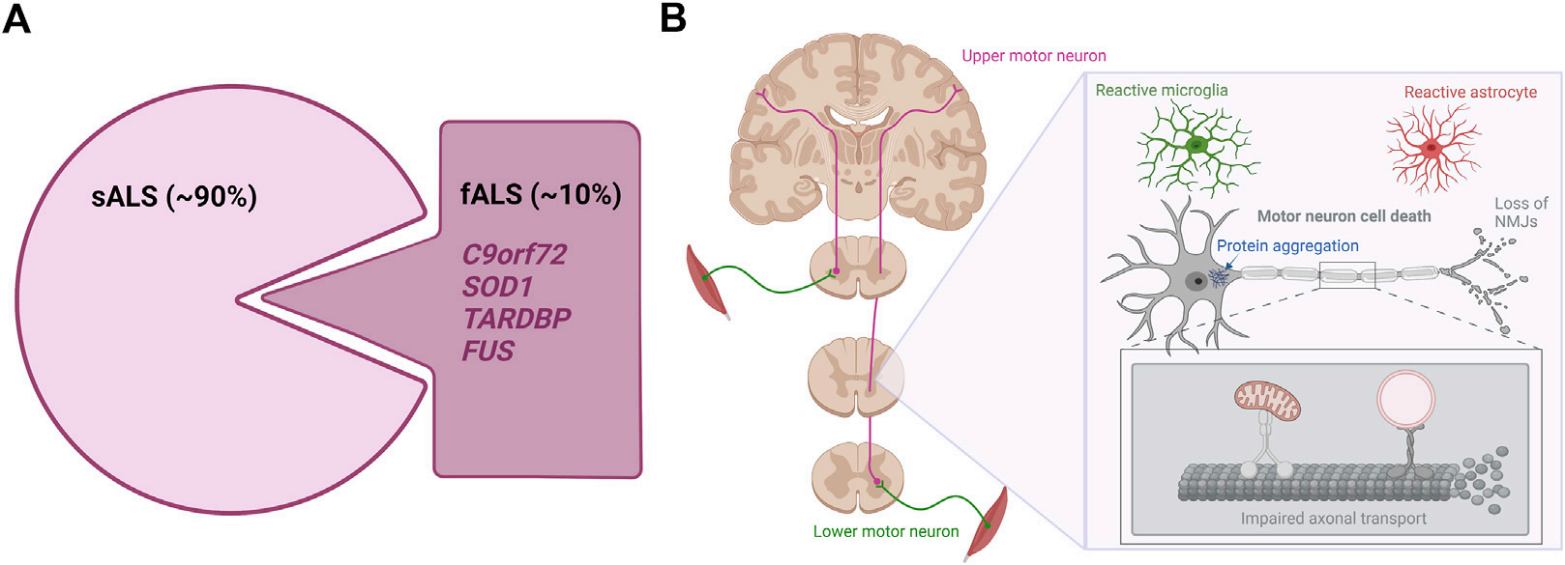
The genetics and hallmarks of ALS. **(A)**. In 90% of cases, ALS is a sporadic disease with no family history. The remaining 10% are caused by familial mutations mainly found in the *C9orf72*, *SOD1*, *TARDBP*, and *FUS* genes **(B)**. ALS affects the upper and lower motor neurons in the brain and spinal cord. Currently hallmarks of ALS disease include motor neuron cell death, protein aggregation, distal motor neuron-muscle disconnection/NMJ loss, axonal transport impairments and reactive gliosis. Figure is created with Biorender.com.

Amyotrophic lateral sclerosis (ALS) is the most common motor neuron disorder in adults. The annual incidence rate ranges from 0.6–1.8 per 100.000 people, while the annual prevalence rate is around 4-8 per 100.000 persons with a higher occurrence in males compared to females (Adamczyk et al., 2021; Agbas et al., 2021). The number of cases varies demographically and a higher prevalence is predominately found in the Western parts of the world (Adamczyk et al., 2021). ALS causes selective and progressive loss of upper motor neurons present in the motor cortex of the brain and lower motor neurons from the brainstem and the ventral horn of the spinal cord. The loss of the upper motor neurons results in spasticity and hyperreflexia, while degeneration of lower motor neurons leads to spontaneous muscle twitching or fasciculations and ultimately to muscle atrophy and weakness (Brown and Al-Chalabi, 2017). Although the clinical presentation is variable, for the majority of patients symptoms usually start at the periphery affecting distal limbs such as hands and feet, but will soon spread to include larger muscle groups, eventually rendering patients wheelchair bound (Adamczyk et al., 2021). The disorder has a rapid disease progression which limits median survival after symptom onset to 2–5 years mostly due to respiratory failure (Masrori and Van Damme, 2020). ALS is a familial disease in 10% of cases (Figure 1A) and in the majority of familial ALS (fALS) the causative gene is known (Renton et al., 2014). The inheritance pattern is usually autosomal dominant, and mutations in the *superoxide dismutase 1* (*SOD1*) gene, in the *TAR DNA binding protein* (*TARDBP*) gene or in the *FUS RNA binding protein* (*FUS*) gene are important genetic causes of ALS (Al-Chalabi et al., 2012). The more recently discovered hexanucleotide repeat expansions in the C*hromosome 9 open reading frame 72* (*C9orf72*) gene are by far the most common genetic causes of ALS as these repeat expansions instigate 30%–50% of fALS (DeJesus-Hernandez et al., 2011; Renton et al., 2011; Van Blitterswijk et al., 2012). In 90% of cases, ALS is a sporadic disease clinically indistinguishable from familial forms, but without a familial inheritance pattern. The etiology of sporadic ALS (sALS) remains elusive, but it is thought to arise from a combination of aging, as well as largely undetermined genetic and environmental factors (Al-Chalabi and Hardiman, 2013; Shatunov and Al-Chalabi, 2021). Interestingly, some risk factors such as history of head trauma, electrocution, lead expose and smoking have been associated with a higher chance of developing ALS (Wang et al., 2017; Andrew et al., 2021), but further research is needed to confirm this. Although ALS is considered an adult-onset disease with an average age of onset around middle-to-late 50s, ALS presents itself rather heterogeneous in terms of age of disease onset. As a result, both juvenile and geriatric cases exist (Shang and Huang, 2016). Currently no effective treatment is available for ALS. Despite decades of research into the complex pathophysiology of ALS, dozens of compounds have failed to show any significant impact on disease during clinical trials despite promising preclinical results. Three drugs have been approved by the Food and Drug Administration (FDA); Riluzole, Edaravone and AMX0035 (Petrov et al., 2017; Paganoni et al., 2020; 2021), while only Riluzole is approved by the European Medicines Agency (EMA). Of the three drugs, Riluzole appears to have the strongest albeit limited effect on survival, and most treatment measures therefore rely on symptomatic and palliative care in order to improve the quality of life of patients (Agbas et al., 2021).

Despite being a complex disorder, certain pathological hallmarks exist (Figure 1B). Apart from the striking motor neuron death evident in all ALS cases, abnormal protein inclusions are found in the cytoplasm of surviving motor neurons in the vast majority of ALS patients. Especially the TDP-43 proteinopathy is one of these hallmarks, as 97% of all ALS cases present histologically with TDP-43-positive inclusions independent of *TARDBP* mutations (Neumann et al., 2006; Tziortzouda et al., 2021). These inclusions are not limited to the motor neurons, but are likewise found in glial cells throughout the brain and spinal cord emphasizing a broader involvement of different cell types in ALS (Tziortzouda et al., 2021). Additionally, signs of ALS emerge when connections between lower motor neurons and muscle fail. Several studies in animal models and patients show how nerve terminals and neuromuscular junctions (NMJs) are partially degraded in early stages of the disease, while the motor neuron cell bodies in the spinal cord are mostly intact (Fischer et al., 2004; Nair et al., 2010; Walker et al., 2015; Tallon et al., 2016; Martineau et al., 2018; So et al., 2018; Picchiarelli et al., 2019). Upon axonal retraction, the motor neuron initially compensates by sprouting and collateral re-innervation by new axons. However, when the disease progresses, the motor neuron is no longer able to continuously compensate for the retracting axons and it finally dies (Robberecht and Philips, 2013). Symptoms will only occur when large populations of motor neurons are affected, resulting in weakness of muscle groups. Recent findings suggest that axonal transport deficiencies are a common mechanism in ALS as impaired axonal transport of mitochondria, mRNA and endosomes in motor neurons harbouring mutations in *FUS*, *TARDBP* and *C9orf72* has so far been demonstrated (Alami et al., 2014; Guo, et al., 2017a; Fazal et al., 2021; Fumagalli et al., 2021). Since motor neurons have very long axons, they are more susceptible to axonal retraction and axonal transport dysfunctions than other neurons, further explaining the selective vulnerability in ALS pathogenesis (Robberecht and Philips, 2013). Finally, abnormal glial activation (popularly defined as “gliosis”) is extensively found throughout the brain and spinal cord of both fALS and sALS cases (Agbas et al., 2021; Rossi and Cozzolino, 2021), and especially the involvement of astrocytes will be discussed later in this review.

## 2 Astrocytes in health—One cell, many roles

Despite the fact that ALS is considered a motor neuron disorder, it is increasingly recognized that ALS does not only affect this subpopulation of neurons, but in fact also has a striking influence on other neuronal subtypes as well as on the glial population including astrocytes. Astrocytes are one of the most abundant glial cell types in the central nervous system (CNS) as the glial population is composed of approximately 20%–40% astrocytes (Allen and Lyons, 2018; Verkhratsky and Nedergaard, 2018). This cell type harbours great heterogeneity and is typically organized in highly specialized cellular-level domains, which are focused on the homeostatic needs of its residing region (Verkhratsky and Nedergaard, 2018). The domain diameter varies from <100 to >400 μm (Oberheim et al., 2009), and each astrocytes’ anatomical space is generally non-overlapping, although some interaction through gap junctions between neighbouring domain astrocyte processes is documented at the territorial peripheries (Bushong et al., 2002; 2004). As such, this domain formation could be a reason why atrophy due to neurodegeneration is often seen in specific areas of the brain and it has been hypothesized that neurodegeneration primarily affects astrocytes, which then causes a subsequent spreading to neurons through gap junctions (Sica et al., 2016). Whether this is also the case for ALS is debated.

In order to understand the astrocytic involvement in disease, we first have to understand their role in health. Although astrocytes have been recognized for their important roles in the CNS for decades, research continuously reveals their remarkable functional complexity. Astrocytes are found to be crucial mediators of neurotrophic support, neurotransmitter regulation, synapse plasticity and activity modulation, and for the structural integrity of the brain and spinal cord (Verkhratsky and Nedergaard, 2018). Besides this, astrocytes facilitate the maintenance of an optimal CNS environment through regulation of the blood brain barrier (BBB), water flux, ion and pH homeostasis, and removal of reactive oxygen species (ROS). Astrocytes likewise contribute to the inflammatory and immune response, as they secrete cytokines, phagocytize and facilitate border formation after injury. In the following section, key astrocyte functions will be described in detail.

### 2.1 Astrocyte-neuron network - Synapse modulation and pruning

**FIGURE 2:**
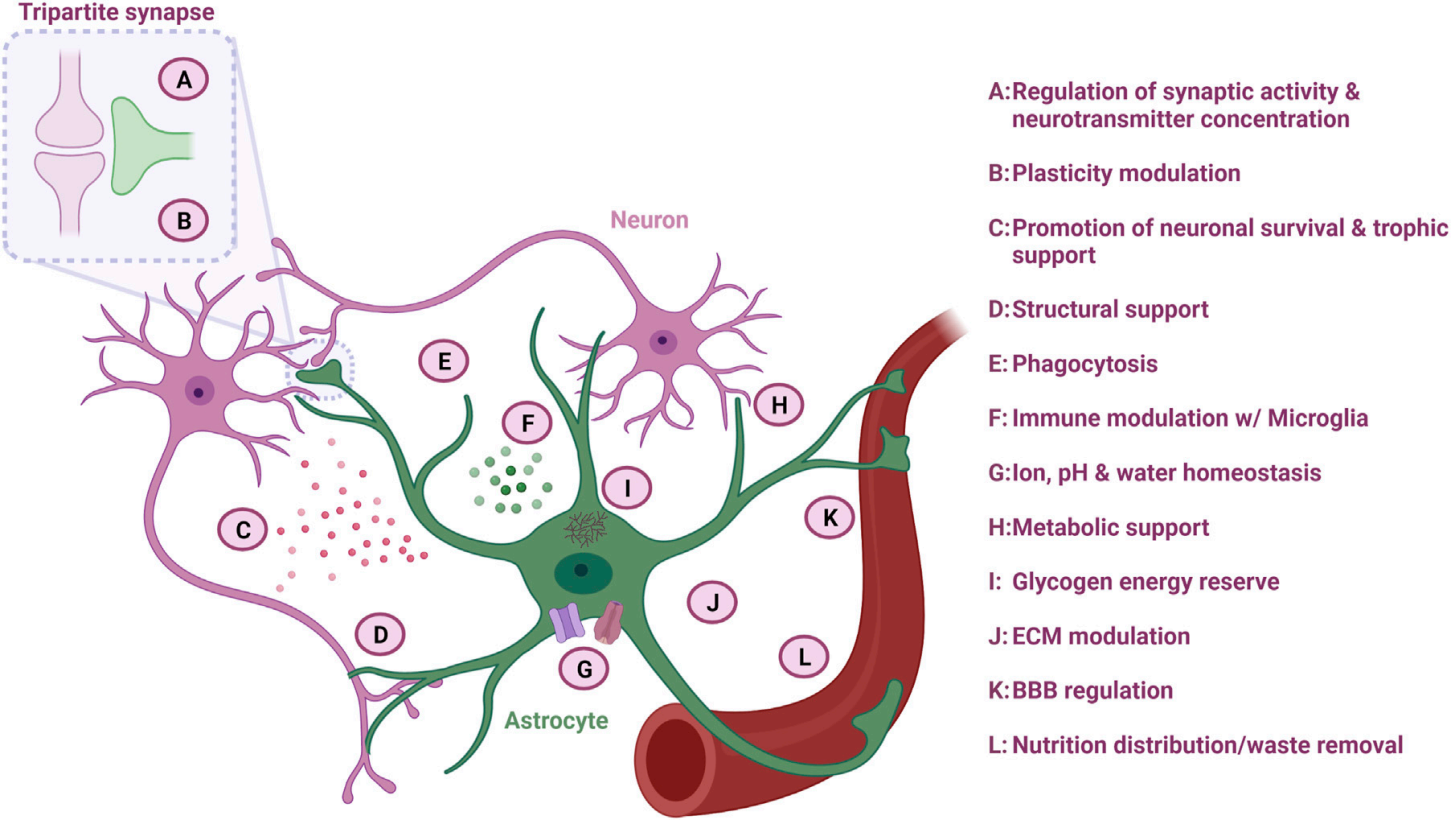
Astrocyte function in the CNS. Astrocytes are multifaceted cells with various important functions overall ensuring optimal neuronal homeostasis. Figure created with Biorender.com.

Astrocytes modulate neuronal synapse plasticity and function through numerous mechanisms. In order to engage with the neuronal network, astrocytes extend their endfeet to form perisynaptic processes (PAPs), which ensheath neuronal synapses and form the so-called “tripartite synapse” (Figure 2). The degree of synapse enwrapping varies between brain regions and depends on synapse morphology. However, it is estimated that at least half of all CNS synapses are covered with astrocytic PAPs (Verkhratsky and Nedergaard, 2018). The astrocytic synapse-enveloping ensures removal of neurotransmitters such as glutamate from the interstitial space through receptor modulation, which counteracts neurotransmitter accumulation and subsequent excitotoxicity (Allen and Eroglu, 2017).

Another mechanism of synapse modulation is through secretion of certain molecules, which is especially important during synaptogenesis. Astrocytes secrete proteins and cytokines such as hevin (SPARCL1), thrombospondins (THBS1/2), glypicans (GPC4/6), transforming growth factor β1 (TGF-β1) and brain-derived neurotrophic factor (BDNF), which through transsynaptic adhesion proteins or receptor modulations promote the formation and maturation of excitatory synapses (Vainchtein and Molofsky, 2020; Liu et al., 2021). TGF-β1 has a dual function as this cytokine likewise promotes the formation of inhibitory synapses in the CNS (Liu et al., 2021). In addition, tumor necrosis factor-α (TNF-α) secreted from astrocytes also plays an important role as it increases the α-Amino-3-hydroxy-5-methyl-4-isoxazolepropionic acid (AMPA) receptors on excitatory synapses, while it downregulates γ-aminobutyric acid (GABA) receptors on inhibitory synapses, overall increasing neuronal activity (Allen and Eroglu, 2017). To negatively regulate synaptogenesis, astrocytes also secrete signals such as SPARC in order to balance the process (Allen and Lyons, 2018).

Synaptic pruning is dependent on neuronal activity (Liu et al., 2021), and astrocytes are also involved in this process (Figure 2). Through direct activation of astrocytic phagocytic receptors Multiple EGF Like domains 10 (MEGF10) and MER proto-oncogene, tyrosine kinase (MERTK) on the astrocyte surface, astrocytes can degrade superfluous synapses in order to improve the precision and efficiency of neuronal networks (Allen and Lyons, 2018). In addition, astrocytes can indirectly modulate synapse pruning by secreting interleukin-33 (IL-33) or by activating the classical complement cascade and C1q expression through TGF-β secretion, which subsequently promotes selective microglia phagocytosis of the synapses destined for degradation (Vainchtein et al., 2018; Liu et al., 2021). As a consequence, astrocytes collaborate with microglia in the dynamic modulation of neuroplasticity, which is especially important for learning and memory.

### 2.2 Ion homeostasis

Astrocytes control the ion homeostasis through multiple ion channels, which is important for maintaining synapse function (Figure 2) (Sibille et al., 2014; Allen and Eroglu, 2017). By regulating the potassium concentration in the extracellular space through clearance *via* potassium channels such as the main K_ir_4.1 type, astrocytes control whether neurons become depolarized after an action potential and thereby affects their ability to fire a signal again (Allen and Lyons, 2018). Especially in the spinal cord, astrocytes possess a large amount of K_ir_4.1 channels, which are important to accommodate the firing rate of large fast motor neurons (Allen and Lyons, 2018; Kelley et al., 2018). Astrocyte can also collaborate with other glial cells such as oligodendrocytes in the formation of a glial syncytium, which controls the potassium concentration and subsequent neuronal activity (Allen and Lyons, 2018).

### 2.3 Structural support and water homeostasis

One of the first recognized functions of astrocytes is their importance in structural support (Figure 2) (Verkhratsky and Nedergaard, 2018). Astrocytes are considered crucial for the integrity of the stroma in the CNS and secrete molecules such as proteoglycans, which are central for the extracellular matrix (ECM) structure within the synaptic cleft and surrounding synapses (Figure 2). This ECM structure ensures the capturing of nutrients and growth factors, as well as buffers the concentration of neurotransmitters to maintain acceptable levels by acting as a diffusion barrier (Liu et al., 2021). Astrocytes also form the glia limitans layer of the pia mater of the CNS through sheets, which contains aquaporin-4 (AQP4) water channels. Through these channels, astrocytes are able to regulate influx and efflux of water within the CNS, and additionally facilitate the glymphatic flow into the perivascular space, which ensures removal of excess fluid and metabolic waste products (Figure 2) (Verkhratsky and Nedergaard, 2018).

### 2.4 Metabolic homeostasis

Neurons consume a vast amount of energy, and therefore rely on continues supply of energy substrates (Figure 2). In order to accommodate this need, astrocytes provide neurons with energy substrates through one of the key mechanisms: the astrocyte-neuron lactate shuttle. Neurons primarily use oxidative metabolism of glucose to produce large amounts of adenosine triphosphate (ATP), while astrocytes predominantly convert glucose to lactate *via* aerobic glycolysis resulting in a low ATP production (Verkhratsky and Nedergaard, 2018). The lactate is transferred to adjacent neurons *via* monocarboxylate transporters (Allen and Lyons, 2018), where the neuronal mitochondria use the lactate in the oxidative cycle for ATP production (Verkhratsky and Nedergaard, 2018). In addition, astrocytes can shuttle lactate from the blood stream directly to neurons (Verkhratsky and Nedergaard, 2018). Astrocytes are also an important player in the glycolytic pathway, as they are the sole processors of brain glycogen (Figure 2) (Verkhratsky and Nedergaard, 2018). During sleep, glycogen is stored, while activity causes glycogen release and metabolism, providing further energy for the cells (Verkhratsky and Nedergaard, 2018). Additionally, astrocytes are the main synthesizers and suppliers of apolipoprotein E (ApoE), and they ensure a sufficient transport of cholesterol to the neurons in the CNS *via* ApoE receptors (Liu et al., 2021). Cholesterol is an important lipid in membranes and is also essential for the function of glutamatergic neurons as it increases the amount of vesicles and their release probability in the presynapse (Allen and Eroglu, 2017).

### 2.5 Blood brain barrier

Astrocytes are the gatekeepers of the CNS as they maintain the BBB through close interaction with the vasculature (Figure 2). As such, the capillary endothelial cells, which are wrapped by pericytes and the basal lamina, are closely surrounded by astrocytic endfeet processes (Harada et al., 2016). This ability of astrocytes to contact both vessels and neurons facilitates the shuttle of nutrients from the capillaries to the neurons and waste product from the neurons back to the blood stream. Astrocytes are also important in the development of the BBB, as they aid the alignment of endothelial cells and pericytes (Harada et al., 2016). Through the calcium-dependent release of vasodilators or vasoconstrictors such as prostanoids and arachidonic acid, astrocytes can control the blood flow depending on the neuronal energy demand (Liddelow and Barres, 2015).

### 2.6 Communication

In contrast to neurons, astrocytes are not able to project excitability through electrical action potentials, but rely on calcium waves to communicate (Liu et al., 2021). Between individual astrocytes and intracellularly, calcium transients move along processes through the Soma *via* calcium-permeable receptors and ion channels. Although calcium stores within the astrocytes’ endoplasmic reticulum account for a large part of the calcium communication, transport of extracellular calcium into the cells is also mediated by glutamate channels made of AMPA and N-methyl-D-aspartate (NMDA) receptors or voltage-gated calcium channels (Liu et al., 2021). Especially NMDA receptors are important for astrocytic-neuronal communication, as they are required for synapse formation and proper dendritic morphogenesis during development (Harada et al., 2016). In addition, gap junctions composed of connexin-43 (Cx43), which is the predominant connexin protein in astrocytes, allow transport of calcium between adjoining astrocytes (Harada et al., 2016).

Astrocytes express multiple receptors, which are responsive to a large variety of neurotransmitters. Many of these receptors are G protein-coupled, which activates an intracellular signal through calcium (Allen and Lyons, 2018). As such, astrocytes are able to distinguish between glutamatergic and cholinergic synaptic activity through the ability of the receptors to evoke a unique calcium response. Different types of calcium waves exist and the uptake requirements of extracellular calcium is dependent on the subpopulation of astrocytes (Clarke et al., 2021). Most transients are found at the processes, which correlates with the ability of increased calcium concentrations to release astrocytic transmitters such as glutamate, D-serine and ATP, which subsequently can regulate neuronal excitability and synaptic plasticity (Liu et al., 2021). Astrocytes also secrete GABA and glycine, which act as inhibitory transmitters in the brain and spinal cord, respectively (Verkhratsky and Nedergaard, 2018). These “gliotransmitters” are primarily released *via* vesicular exocytosis or through specific transporters and hemichannels.

### 2.7 Reactive astrocytes

Under physiological conditions, non-activated astrocytes are primarily tissue embedded and non-motile, firmly devoted to their catering to neuronal homeostasis within their specific domain (Vainchtein and Molofsky, 2020). Upon injury, disease or infection, some astrocytes become hypertrophic, extend their processes and together with microglia initiate an immune response through cytokine and interleukin secretion prompting a strong collective fight against the harmful agent (Figure 2) (Sica et al., 2016). This inflammatory activation contributes to the remodelling of the BBB, which can allow influx and guidance of peripheral leukocytes further enhancing the immune response (Sica et al., 2016). Activated astrocytes are termed “reactive.” As a consequence, reactive astrocytes might down-prioritize their homeostatic obligations, leaving the neurons under less surveillance (Sica et al., 2016). As such, abnormal and/or chronic astrocyte reactivity observed in neurodegenerative disorders such as ALS likely plays a central role in neuronal distress and cell death.

At least two broad subtypes of reactive astrocytes have been identified, however more are likely present (Sofroniew, 2020). Upon acute focal injuries such as trauma, ischemia, local infection or toxic accumulation, one type of barrier-forming reactive astrocytes will migrate towards the site of injury, proliferate and commence the formation of a physical barrier encircling and containing the damage (Sofroniew, 2020; Vainchtein and Molofsky, 2020). The astrocytic border formation is highly enriched in glial fibrillary acidic protein (GFAP) and also contains large numbers of activated microglia, which remove debris through phagocytosis (Vainchtein and Molofsky, 2020). In the past, this barrier has extensively been referred to as a “glial scar.” However, this nomenclature is now considered somewhat outdated due to the clear difference from scar tissue formed in other tissues (Sofroniew, 2020). The second identified subtype of reactive astrocytes are usually non-proliferative and retain their position and cellular interaction with the CNS, but still respond locally to injury or disease through functional and sometimes morphological changes (Sofroniew, 2020). These cells are shown to undergo hypertrophy within their individual domains, but still maintain their intercellular connections with neurons, glia and the vasculature (Sofroniew, 2020). Emerging studies reveal how this subtype of astrocytes might modulate synapses or alter their homeostatic support. However, little is known about their complex functional alterations in response to injury. Nevertheless, their response is thought to be context-dependent and modulated based on their current state and the incoming reactivity triggers (Sofroniew, 2020). Therefore, it is favourable to discuss both the myriad of potential reactive astrocyte subtypes, as well as the concept of variable reactive states within each subtype. This can be influenced by factors like disease type and its progression over time (Sofroniew, 2020). Importantly, physiologically adaptive reactive astrocytes should not be confused with disease-induced dysfunctional astrocytic reactivity, which contributes to cellular stress and neurodegeneration through cell-autonomous abnormalities. In contrast, the reactive astrocytes constitute a physiological response to injury in order to protect neuronal tissue, re-establish homeostasis and preserve neurological function (Sofroniew, 2020).

## 3 Amyotrophic lateral sclerosis is a non-cell autonomous disease—The role of astrocytes

In the unravelling of ALS pathogenesis, the primary focus has been mainly on motor neurons. Although, the motor neurons are inarguably the most susceptible or vulnerable cell type in motor neuron disease, hence the name, several studies examining the contributions of astrocytes have been conducted the last couple of decades with remarkable results. And the interest is still growing.

Observations of “bizarre,” “abnormal” or reactive astrocytes as well as “astrocytosis” and “astrogliosis” have been described in several ALS case reports since the 1970’s (Smith et al., 1975; Ghatak and Nochlin, 1982; Kamo et al., 1987; Kushner et al., 1991; Murayama et al., 1991; Nagy et al., 1994; Schiffer et al., 1996). These reports relied primarily on GFAP expression and described increased astrocyte numbers and abnormal morphology throughout both brain and spinal cord tissue. It is now established that astrocyte reactivity is a prominent hallmark in ALS patients (Haidet-Phillips et al., 2011; Qian et al., 2017; Guttenplan et al., 2020), and that the number of reactive astrocytes is shown to correlate with disease progression (Hall et al., 1998).

Most experimental *in vivo* results have been obtained using mutant *SOD1* transgenic mouse models (Gurney et al., 1994; Wong et al., 1995; Bruijn et al., 1997). This model, available since the 1990’s, shows motor dysfunction and motor neuron loss causing a life expectancy varying between 5 and 15 months depending on the copy number and the specific *SOD1* mutation. Initially, it was shown that neither (motor) neuron- nor astrocyte specific expression of mutant SOD1 results in motor dysfunction, indicating an interplay between neurons and non-neuronal cells in the disease progression (Gong et al., 2000; Pramatarova et al., 2001; Lino et al., 2002). This interplay was confirmed in chimeric mouse models, where mutant *SOD1*-containing motor neurons survived longer when surrounded by wild type non-neuronal cells (Clement et al., 2003). A later study reported that neuron-specific expression of human *SOD1* did in fact cause degeneration and paralysis in mice, but importantly only in homozygous transgenic mice (Jaarsma et al., 2008). They too did not report any pathology mediated by heterozygous mutant *SOD1* expression as in the other studies. Interestingly, transplantation of astrocyte precursor cells harbouring a human *SOD1* ^*G93A*^ mutation into wild type rodent spinal cords generated mutant reactive astrocytes, which were sufficient to induce motor neuron death, forelimb motor and respiratory dysfunction in addition to reduced glutamate transporter 1 (GLT-1) expression (Papadeas et al., 2011). These findings were in line with previous observations, where SOD1-containing inclusions were extensively found in astrocytes but not in neurons during the initial stages of the disease and that these inclusions became more abundant during disease progression, in addition to increased GFAP expression and loss of GLT-1 (Bruijn et al., 1997). In transgenic *SOD1* ^*G93A*^ mice, degeneration of astrocytes residing around spinal motor neurons was found to precede the motor symptoms caused by neuronal cell death, while “astrocytosis” continually progressed during the course of the disease (Rossi et al., 2008). Astrocyte involvement is not only evident in *SOD1*-ALS, as a recent study demonstrated how sALS patient induced pluripotent stem cell (iPSC)-derived astrocytes caused motor neuron degeneration, NMJ denervation and motoric deficits, when transplanted into the spinal cord of immune-deficient mice (Qian et al., 2017). Oppositely, transplantation of healthy astrocyte precursors into spinal cords of human *SOD1* transgenic rats resulted in reduced microglial reactivity, extended disease duration and survival, attenuated motor neuron loss and slowed the decline in motor and respiratory functions (Lepore et al., 2008). In addition, astrocyte-specific deletion of mutant *SOD1* delayed microglial activation, slowed down the disease progression in mice substantially (Yamanaka et al., 2008; Wang et al., 2011) and restored GLT-1 levels (Wang et al., 2011).

*In vitro* coculture experiments have likewise confirmed the role of astrocytes in ALS. Cocultures between murine embryonic stem cell (ESC)-derived motor neurons harbouring a human *SOD1* ^*G93A*^ mutation with primary or ESC-derived *SOD1* ^*G93A*^ mutant glia, like astrocytes, showed glial toxicity affecting the motor neuron survival (Di Giorgio et al., 2007). These effects were also present in the gene expression changes, supporting the notion that cultured motor neurons and glia affect each other (Phatnani et al., 2013). Importantly, the gene expression profiles showed concordance with *in vivo* spinal cord tissue, proving the validity of cocultures in recapitulating important aspects of ALS pathogenesis (Phatnani et al., 2013). Similarly, healthy murine (m) or human (h) ESC-derived motor neurons showed survival impairments when directly cocultured with or receiving conditioned media from primary human or rodent *SOD1-*mutant glia (Nagai et al., 2007; Di Giorgio et al., 2008; Marchetto et al., 2008). These astrocyte-mediated toxic effects were not only apparent in *SOD1*-ALS, but were also documented in cocultures between astrocytes generated from sALS *post-mortem* tissue and wild type m- or hESC-derived motor neurons as well as through sALS astrocyte conditioned media (Haidet-Phillips et al., 2011; Re et al., 2014). Relatedly, directly reprogrammed fibroblasts into astrocytes from *C9orf72*-, *SOD1*-and sALS patients showed non-cell autonomous toxicity on wild type mESC-derived motor neurons, as motor neuron survival was greatly impaired due to the presence of the ALS astrocytes (Meyer et al., 2014). Despite the multiple overlaps between fALS and sALS presented above, TDP-43-ALS remains controversial. Some studies using astrocytic mutant TDP-43 overexpression or TDP-43-ALS (h)iPSC-derived astrocytes did not report any pathologic effects on motor neurons neither *in vitro* nor *in vivo* (Haidet-Phillips et al., 2013; Serio et al., 2013). In contrast, other studies did in fact show astrocyte-specific mutant TDP-43-mediated motor neuron death, muscle denervation, and paralysis in addition to astrocyte glutamate transport GLT-1 and Glutamate/aspartate transporter (GLAST) depletion (Tong et al., 2013; Rojas et al., 2014). These conflicting results suggest that the toxic astrocytic effects might not apply to all forms of ALS, or that caution must be implemented when comparing results obtained from different models. Nevertheless, based on these *in vivo* and *in vitro* results, it is difficult to argue against a role of astrocytes in ALS pathology and progression. However, the question is; how much do we know so far about the mechanism(s) involved?

### 3.1 Loss of support mechanisms

**FIGURE 3:**
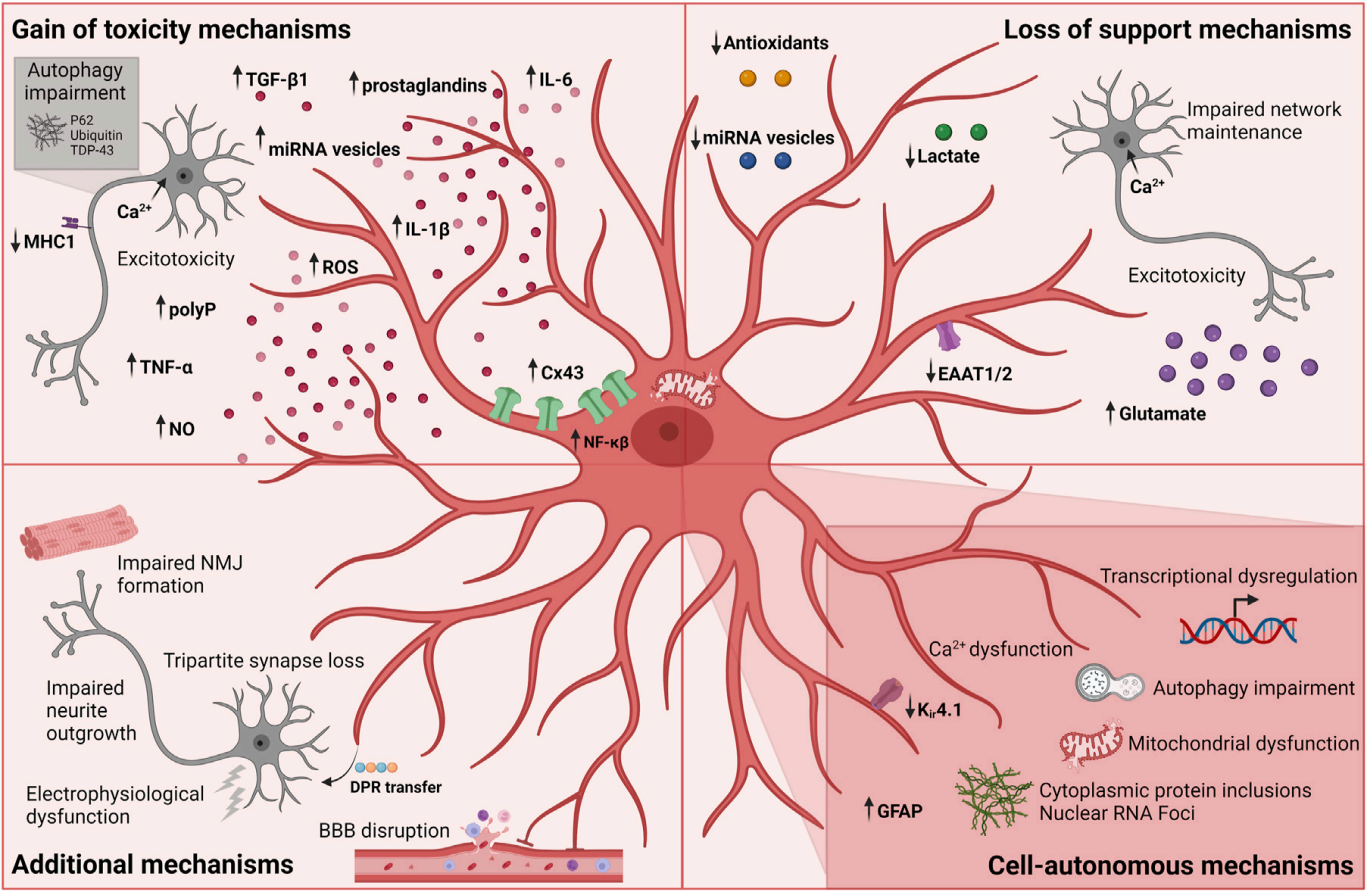
Currently known mechanisms in astrocyte-mediated ALS pathology. Each mechanism is divided into broader categories of gain of toxicity, loss of support, additional and cell-autonomous mechanisms. Figure is created with Biorender.com.

The hypothesis of excitotoxicity being a potential pathologic hallmark of ALS emerged with the report of increased glutamate concentrations in sALS patient cerebrospinal fluids in early disease progression together with the discovery of reduced glutamate transport a few years later (Van Den Bosch et al., 2006). As mentioned above, downregulation or loss of GLAST/GLT-1 transport in astrocytes has been extensively shown in *in vivo* models, and is likely correlated with abnormal levels of extracellular glutamate, causing excessive neuronal firing, abnormal influx of neuronal calcium and ultimately excitotoxicity (Figure 3) (Fritz et al., 2013). In addition, *SOD1*-ALS astrocyte-mediated dysregulation of the glutamate receptor 2 (GluR2) subunit of AMPA receptors on motor neurons resulted in increased excitotoxic vulnerability (Van Damme et al., 2007). As a result, efforts went into drug development targeting this mechanism and in 1995 Riluzole was approved for ALS treatment (Bensimon et al., 1994). Riluzole is an anti-glutamatergic agent with anti-excitotoxic properties and inhibits postsynaptic NMDA and AMPA receptors to glutamate as well as its release (Doble, 1996). Life expectancy is prolonged with several months for ALS patients receiving Riluzole treatment, while providing no relieve of symptoms (Jaiswal, 2018). Despite being the only available drug for all ALS patients with a proven effect on survival, its limited therapeutic influence might indicate that ALS pathogenesis is driven by more than one mechanism. Abnormal levels of glutamate clearly have a toxic effect on motor neurons and this could be due to the higher vulnerability of motor neurons to excitotoxicity (Van Den Bosch et al., 2006) in combination with the loss of glutamate transporters and thereby lack of astrocytic support of the homeostatic environment.

Another line of studies explores a similar support mechanism within the astrocyte metabolism: the lactate shuttle. In the context of *SOD1*-mutations, results from both *in vivo*, *in vitro* and ALS patient material showed a decrease in astrocytic intracellular and extracellular lactate levels as well as impairment of the astrocyte lactate efflux transporter (Figure 3) (Ferraiuolo et al., 2011; Madji Hounoum et al., 2017). In addition to the apparent lack of lactate shuttling, low lactate levels could also be caused by a decrease in lactate production due to impaired aerobic glycolysis induced by the downregulation of astrocytic nicotinamide adenine dinucleotide (NAD^+^) (Madji Hounoum et al., 2017).

Astrocytes have also been shown to harbour mitochondrial dysfunction and oxidative stress resulting in motor neuron degeneration (Cassina et al., 2008; Birger et al., 2019). Mitochondrial dysfunctions including compromised oxygen consumption and decreased membrane potential were observed in rat *SOD1* ^*G93A*^ astrocytes, which caused astrocyte-induced motor neuron death (Cassina et al., 2008). Interestingly, this toxicity could be rescued by treatment with mitochondria-targeted antioxidants suggesting a loss of astrocytic antioxidant secretion (Figure 3). Similarly, hiPSC-derived astrocytes from *C9orf72*-ALS patients showed increased oxidative stress as well as a lower secretion of antioxidants (Birger et al., 2019) further confirming this hypothesis. Upon culturing hESC-derived motor neurons in *C9orf72*-ALS astrocyte conditioned media, a similar increase in ROS levels was found in addition to impaired motor neuron survival. To therapeutically target oxidative stress in ALS, Edaravone was used as a free radical scavenger. However, Edaravone shows a limited effect on disease progression and only in a subset of ALS patients (Cho and Shukla, 2020; Witzel et al., 2022). This potential loss-of-support mechanism mediated through the astrocyte secretome correlates with the lack of microRNA (miRNA)-containing vesicles (Figure 3) (Varcianna et al., 2019; Gomes et al., 2022). *C9orf72*-ALS astrocytes were shown to have impaired extracellular vesicle formation leading to dysregulated levels of miRNAs (Varcianna et al., 2019). Especially lower levels of secreted miR-494-3p correlated with improper neurite network maintenance, and by restoring miR-494-3p levels, neurite length was restored and motor neuron survival improved (Varcianna et al., 2019). Low levels of mir-494-3p was also found in sALS *post-mortem* cortico-spinal tracts suggesting a more general mechanism in ALS (Varcianna et al., 2019).

### 3.2 Toxic gain of function mechanisms

Studies using astrocyte-conditioned medium also tested the hypothesis whether astrocytic release of soluble factors could mediate toxic gain of function reactions (Nagai et al., 2007; Di Giorgio et al., 2008; Rojas et al., 2014). Multiple factors were proposed including the release of cytokines such as TNF-α, interleukin 6 (IL-6) and interleukin 1β (IL-1β) as well as inorganic polyphosphate (polyP), miRNA-containing extracellular vesicles, prostaglandins, nitric oxide (NO), and ROS (Figure 3) (Nagai et al., 2007; Di Giorgio et al., 2008; Marchetto et al., 2008; Mishra et al., 2016; Kia et al., 2018; Chen et al., 2019; Varcianna et al., 2019; Lee et al., 2020; Arredondo et al., 2022; Baofengfeng et al., 2022; Gomes et al., 2022; Jensen et al., 2022; Stoklund Dittlau et al., 2023). Additionally, upregulation of inflammatory genes targeting proinflammatory cytokines, chemokines and components of the complement cascade have been reported in both fALS and sALS (Di Giorgio et al., 2008; Marchetto et al., 2008; Haidet-Phillips et al., 2011; Stoklund Dittlau et al., 2023) in addition to inflammatory NF-κβ pathway upregulation in sALS astrocytes (Haidet-Phillips et al., 2011; Gomes et al., 2019). The involvement of the NF-κβ pathway in astrocyte ALS pathology supports the notion that not only microglia are implicated in the neuroinflammatory response. Interestingly, although NF-κβ upregulation is found in astrocytes, astrocytic inhibition of the pathway did not rescue motor dysfunction or affect disease progression in *SOD1* mice (Crosio et al., 2011; Frakes et al., 2014), while having the opposite and beneficial effect on microglia-induced motor neuron toxicity (Frakes et al., 2014). These results argue that microglia are highly reactive and toxic through NF-κβ pathway activation, while astrocytes likely contribute to ALS pathogenesis through a variety of mechanisms. Interestingly, a follow-up study showed how specific NF-κβ suppression in microglia, in combination with SOD1 reduction in motor neurons and astrocytes prolonged the lifespan and motor function in mice (Frakes et al., 2017). This does not only confirm the complex interplay between cell types and their combined influence on ALS pathogenesis, but also the additive effect of targeting multiple cell types in therapy development. In line with these results, overexpression of wild type *FUS* also provoked an inflammatory response in astrocytes causing motor neuron toxicity and proinflammatory microglial activation (Ajmone-Cat et al., 2019). Similarly, astrocytic NF-κβ activation drove microglial proliferation and leukocyte infiltration in *SOD1* ^*G93A*^-mice (Ouali Alami et al., 2018). In contrast, another study reported how microglial activation preceded disease onset in *hSOD1* ^*G93A*^ mice, while an increase in astrocytic GFAP-expression appeared later at symptom onset (Gerber et al., 2012). Although these results were acquired from overexpression models, they target the ongoing and interesting “chicken and the egg” discussion whether astrocytes activate microglia or *vice versa* (Liddelow et al., 2017).

Excitotoxicity might also be mediated through a toxic gain of function independent of glutamate levels. Recently, it was shown that astrocyte-secreted polyP mediates motor neuron toxicity in cultures (Arredondo et al., 2022). PolyP is proposed to act as a gliotransmitter by regulating neuronal excitability through ion channel modulation resulting in an increase in action potentials (Stotz et al., 2014). As such, excessive polyP secretion by ALS astrocytes could be linked to excessive influx of calcium causing excitotoxicity in motor neurons (Fritz et al., 2013; Arredondo et al., 2022). Elevated levels of polyP have been found in glial cells from fALS and sALS patient spinal cord sections and in cerebrospinal fluid, further confirming its relevance for ALS (Arredondo et al., 2022).

Astrocyte-induced autophagy impairment has been shown in several studies (Figure 3) (Madill et al., 2017; Tripathi et al., 2017; Allen et al., 2019a). One study showed how ALS-patient iPSC-derived astrocyte conditioned media caused P62 accumulation in HEK293T cells, suggesting an impairment in autophagic flux (Madill et al., 2017). Another study reported how primary reactive mouse astrocytes expressing human *SOD1* ^*G93A*^ caused the formation of cytoplasmic protein inclusions of P62, ubiquitin and TDP-43, as well as axonal phosphorylated neurofilament heavy chain in hESC-derived motor neurons upon prolonged coculture (Tripathi et al., 2017). Interestingly, this effect was found to be independent of genotype but rather relates to reactivity, as traumatic injury-induced reactivity in wild type astrocytes caused similar motor neuron protein aggregations. The protein aggregations were correlated with reactive astrocytic secretion of TGF-β1, which caused a disruption of motor neuron autophagy through the mTOR-pathway (Figure 3). TGF-β1 has been reported as a neuronal pro-survival factor and, as previously mentioned, it is an important astrocytic regulator of synaptogenesis. However, astrocytic TGF-β1 overexpression or prolonged exposure to motor neurons might have detrimental effects. Indeed another study showed how excessive astrocytic TGF-β1 expression inhibited a neuroprotective microglial inflammatory response and enhanced the disease progression (Endo et al., 2015). Overall, the study by Tripathi and colleagues suggests that the reactive state rather than the *SOD1* mutation is driving the pathology in this context.

Abnormal increases in Cx43 expression both at transcription and protein level have been documented in various fALS and sALS *in vitro* and *in vivo* models, as well as in sALS patients (Figure 3) (Almad et al., 2016; 2022; Gomes et al., 2022). As previously mentioned, Cx43 gap junctions and hemichannels mitigate calcium waves in addition to facilitating diffusion of ions, metabolites, miRNAs and various second messengers (Giaume et al., 2010). As a consequence of Cx43 upregulation, increases in astrocytic gap junctional coupling and hemichannel activity led to intracellular and possibly intercellular calcium hyperactivity causing motor neuron excitotoxicity and cell death (Almad et al., 2016; 2022). Interestingly, this toxicity could be counteracted by Cx43 gap junction and hemichannel blockers.

Last but not least, ALS astrocyte-mediated downregulation of the expression of major histocompatibility complex 1 (MHC1) on motor neurons was shown to make motor neurons more vulnerable to astrocyte-induced cell death (Figure 3) (Song et al., 2016).

### 3.3 Additional mechanisms in amyotrophic lateral sclerosis astrocyte-mediated motor neuron pathology

During ALS disease progression, the electrophysiological properties of motor neurons are influenced (Sareen et al., 2013; Wainger et al., 2014; Devlin et al., 2015; Naujock et al., 2016; Guo et al., 2017b), a process also affected by astrocytes (Figure 3). hiPSC-derived *C9orf72*-astrocytes compromised the electrophysiological output of wild type hiPSC-derived motor neurons in a time-dependent manner, despite having no effect on motor neurons death (Zhao et al., 2020). Another recent study discovered how loss of tripartite synapses in *SOD1* ^*G93A*^ mice occurred following early onset of motor deficits (Figure 3) (Broadhead et al., 2022). This tripartite loss was confirmed in *C9orf72*- but not *SOD1*-patient cervical spinal cord *post-mortem* tissue, and might be linked to a loss of astrocyte PAPs (Broadhead et al., 2022). Along the same lines, astrocytes were shown to contribute to the disruption of the BBB, which causes a disturbance of the homeostatic environment and an influx of blood leukocytes (Figure 3) (Garbuzova-Davis et al., 2007a; Garbuzova-Davis et al., 2007b). Additionally, astrocytes were shown to take up toxic *C9orf72* dipeptide repeats (DPRs) from the medium through endocytosis and redistributing them to motor neurons *in vitro*, arguing for possible prion-like protein properties as well as an astrocyte-mediated spreading of neurodegeneration (Figure 3) (Marchi et al., 2022). Interestingly, another study showed how TDP-43 aggregates could propagate from motor neurons to astrocytes, suggesting heterogeneity in spreading mechanisms across the ALS mutation spectrum (Smethurst et al., 2020). Further studies are needed to elaborate on the apparent toxic protein propagation between cell types in ALS.

Whether all these effects are due to a loss-of-support or gain-of-toxicity astrocyte mechanism remains unknown. Recently, we found that *FUS*-ALS hiPSC-derived astrocytes impaired hiPSC-derived motor neuron neurite outgrowth and NMJ formation in a human microfluidic triculture system (Figure 3) (Stoklund Dittlau et al., 2023). These findings correlated with the intriguing observation that mutant astrocytes simultaneously downregulated neuronal support pathways, in addition to upregulating toxic properties. As such, our data argue for a synergistic interplay between both loss-of-support and gain-of-toxicity mechanism in ALS.

### 3.4 Cell-autonomous effects on astrocytes

Besides the clear influence of ALS astrocytes on motor neuron pathology, many studies confirm perturbed intracellular mechanisms in the astrocytes themselves spanning multiple ALS mutations (Figure 3). In addition to the mechanisms mentioned above, transcriptional dysregulation were reported in several studies (Figure 3) (Baker et al., 2015; Sun et al., 2015; Zhao et al., 2020; Taha et al., 2022; Stoklund Dittlau et al., 2023). These findings include disease stage-dependent upregulation of an immune response, lysosomal and phagocytic pathways and downregulation of ion and cholesterol homeostasis in *SOD1* ^*G93A*^ mice astrocytes (Baker et al., 2015; Miller et al., 2018). Similarly, fALS-patient iPSC-derived astrocytes showed transcriptional dysregulations causing a cell-autonomous reactive transformation involving activation of inflammatory pathways, as well as a suppression of homeostatic support, albeit in a heterogeneous mutation-dependent manner (Taha et al., 2022; Ziff et al., 2022; Stoklund Dittlau et al., 2023). In addition, cytoplasmic protein inclusions are present in astrocytes spanning various mutations (Bruijn et al., 1997; Serio et al., 2013; Barton et al., 2020) and iPSC-derived *C9orf72*-ALS astrocytes contain pathogenic nuclear RNA foci (Figure 3) (Zhao et al., 2020). *SOD1*-ALS patient iPSC-derived astrocytes display downregulation of K_ir_4.1 expression (Kelley et al., 2018), and murine *SOD1*-ALS astrocytes were reported to have abnormal intracellular calcium dynamics (Figure 3) (Kawamata et al., 2014). Metabolic screenings on directly from fibroblast-reprogrammed astrocytes of *C9orf72* and sALS patients revealed compromised metabolic flexibility under starvation conditions with defects in adenosine, fructose and glycogen metabolism in addition to impaired mitochondrial substrate transport (Figure 3) (Allen et al., 2019a). In addition, low levels of adenosine deaminase hampered astrocyte adenosine metabolism potentially contributing to motor neuron toxicity (Allen et al., 2019b). This likely correlates with the mitochondrial dysfunctions previously mentioned. Finally, cortical organoid-derived astrocytes from *C9orf72*-ALS patient iPSCs as well as sALS iPSC-derived astrocytes showed perturbed autophagy and P62 accumulations (Figure 3) (Szebényi et al., 2021; Baofengfeng et al., 2022), which argues for a role of autophagy across multiple cell types. Considering the heterogeneous nature of the processes currently known, it is highly likely that many more astrocytic cell-autonomous mechanisms exist in ALS.

### 3.5 Astrocyte-targeted therapy

Astrocyte-targeted treatment might pose a valid therapeutic strategy. Prostaglandin inhibition reduced the toxic effects of glia on motor neuron survival *in vitro* (Di Giorgio et al., 2008), while antioxidant treatment decreased the production of ROS from astrocytes and rescued motor neuron death (Marchetto et al., 2008). Neutralizing antibodies targeting TNF-α alleviated *FUS*-ALS astrocyte-induced motor neuron toxicity (Kia et al., 2018; Jensen et al., 2022), and activation of GLT-1 expression mediated through *ß*-lactam antibiotic administration in *SOD1*-mice resulted in delayed disease progression (Rothstein et al., 2005), although this later failed in a clinical trial. Adeno-associated virus (AAV)-based therapy using miRNAs to suppress human SOD1 expression in astrocytes was shown to preserve muscle innervation, prevent muscle atrophy, and sustain motor performance in *SOD1* ^*G93A*^ mice, although the most prominent effects came from SOD1 silencing in either spinal motor neurons or both cell types with additional rescue of NMJ function, delaying of disease onset and prolonged survival (Dirren et al., 2015; Rochat et al., 2022). Recently, synthetic EphA4 agonists were shown to prevent astrocyte-mediated motor neuron toxicity in astrocyte-motor neuron cocultures in both a *SOD1*-ALS and sALS context (Dennys et al., 2022), while mesenchymal stem cell-derived miRNA-containing extracellular vesicles as well as transfection with miRNA mimics could ameliorate the neurotoxic profile of ALS-astrocytes (Provenzano et al., 2022). Finally, astrocyte replacement strategies with engraftment of healthy (human) astrocyte progenitors into ALS rodent spinal cords have also proven successful in retaining their gene-expression independent of the environment, albeit with conflicting effects on disease progression (Lepore et al., 2008; 2011; Kondo et al., 2014; Haidet-Phillips et al., 2015; Izrael et al., 2018). Whether astrocyte replacement strategies and/or astrocyte-targeted treatments are viable therapy options for ALS patients is an open question.

## 4 Discussion

The role of astrocytes in ALS pathogenesis is complex. Even with the current knowledge, several aspects related to the pathologic process in ALS are not completely understood. The dynamic interplay between loss-of-support and gain-of-toxicity astrocyte functions complements recent findings in Alzheimer’s disease (Jiwaji et al., 2022), which argue for potential common disease mechanisms across multiple neurodegenerative disorders. However, the intricacy of the current knowledge also poses the question whether we are only just scratching the surface? Astrocytes are impressively multifaceted; in their morphology, their location and their function, which likely transfers into a disease context as well.

To dive further into the exact role of astrocytes in ALS and other neurodegenerative diseases, challenges arise with the available models. No model system is perfect, and much insight has inarguably been gained with past and current ALS models. Traditional mice models have been a corner stone in ALS research for many years and extensively bridged the gap between *in vitro* and patient material. However, we have to tread lightly when it comes to the impulse of directly transferring rodent results to a human context, as several reports highlight a clear interspecies variability concerning astrocytes' morphology and function. The glial-to-neuron ratio increases as we climb the evolutionary ladder: from ∼0.05–0.1 in invertebrates, 0.3–0.4 in rodents, 0.5–1.0 in rhesus monkeys and 1.5->2 in humans. It is evident that the number of glia grows with the size of the brain and thus with the higher need for neuronal homeostatic support (Verkhratsky and Nedergaard, 2018). Additionally, some subtypes of astrocytes in the human brain are not found in the rodent brain, which complicates the use of these animal models for studies on human physiology and disease (Verkhratsky and Nedergaard, 2018). Astrocytes also differ greatly in size and complexity when comparing human and rodent cells, as human astrocytes are ∼2,6-fold larger in diameter and process length and 10-fold more ramified than their rodent counterparts (Oberheim et al., 2009; Allen and Eroglu, 2017). In addition, human astrocytes have greater overlap of individual astrocyte domains than rodents, communicate through faster and stronger calcium waves and show a different transcriptional output (Oberheim et al., 2009; Zhang et al., 2016; Li et al., 2021). One astrocyte has contact with 20–120.000 synapses in the mouse brain, whereas a human astrocyte interacts with up to 2 million synapses (Bushong et al., 2002; Oberheim et al., 2009). Remarkably, when engrafting human astrocyte progenitors into immune-deficient mice, human astrocytes retained their morphology and calcium communication, while enhancing the synapse plasticity and learning ability of the chimeric mice. This suggests that species-specificities of astrocytes play a central role in the human cognitive superiority (Han et al., 2013). Importantly, many successful results from ALS drug testing in rodents have failed to translate in clinical trials in humans. One reason for this translational gap could be the timing of treatment, since ALS-mutant mice often are treated before the onset of symptoms, while ALS patients usually receive treatment much later into disease progression. However, it could also be caused by the important species differences between mice and man despite an average genomic overlap of 85% of protein-coding regions and 90% of the expressed genes in astrocytes (Chandrasekaran et al., 2016).

One solution to this species-variability problem is the use of patient material. *Post-mortem* tissue is an obvious and highly valuable resource, but only provide end-stage insights into ALS. On the other hand, newer techniques involving stem cell technology and especially iPSC technology harbour massive potential. From relying on overexpression of human genes in animals, primary (non-)neuronal cultures, and end-stage insight from *post-mortem* tissue, it became possible to take somatic cell biopsies from patients, reprogram these cells into iPSCs followed by differentiation into any desired neuronal or glial cell type in order to model specific patient pathophysiology. As such, researchers now have a tool to model fALS and maybe even more importantly sALS, which had been impossible before. This technique even bypasses the ethical dilemmas connected to the use of hESCs, which has vastly accelerated the stem cell field over the last decade. With the discovery and application of clustered regularly interspaced short palindromic repeats (CRISPR)/Cas9 gene-editing, genetic variability between disease and control lines can be diminished. As described in previous sections, much insight into astrocyte mechanisms in ALS has been gained using stem cell models, and more will likely come. However, despite the clear potential of iPSCs, important limitations remain. These disadvantages include phenotypic variabilities, insufficient maturational state and aging, as well as lack of complexity compared to *in vivo* models (Agbas et al., 2021). To target these limitations, researchers are exploring the use of standardized protocols, genetic- or chemical-induced aging or direct reprogramming, as well as the development of complex *in vitro* cocultures through the use of microfluidic devices and organoids (Meyer et al., 2014; Mertens et al., 2015; Guo et al., 2017b; Agbas et al., 2021; Stoklund Dittlau et al., 2021a; 2021b; Gatto et al., 2021; Fathi et al., 2022; Dennys et al., 2023).

Our general understanding of the underlying disease mechanisms of ALS is derived from years of research using both *in vivo* and *in vitro* models as well as *post-mortem* tissue. Important insights have been gained using these approaches, albeit with limited effect on therapeutic drug development. No cure or effective treatment is currently available for ALS, and it is increasingly acknowledged that this is due to large mechanistic complexity, genetic variability and a translational gap between model and patient. Fortunately, the use of *in vitro* stem cell models has made it easier to obtain mechanistic insight in ALS, and with their growing sophistication, they now propose an important resource for fundamental research as well as for drug discovery. More research is needed to gain the full picture of astrocyte involvement in ALS, but with the increasing interest in the research community and the ongoing improvement of models, there is clear room for optimism. Without a doubt, we should care about astrocytes in motor neuron disease.
